# Real-world experience with CLAIRYG® 50 mg/mL (intravenous immunoglobulin) in children under 12 years with primary immunodeficiency or immmune thrombocytopenia: a post-approval safety study

**DOI:** 10.3389/fped.2023.1260296

**Published:** 2023-10-02

**Authors:** Nizar Mahlaoui, Fanny Fouyssac, Françoise Mazingue, Coralie Mallebranche, Malika Barthez-Toullec, Lamia Denti, Kalaivani Ruhier, Marie-Hélène André-Bonnet, Aude Marie-Cardine, Nathalie Aladjidi, Jean-Louis Stephan

**Affiliations:** ^1^ Pediatric Immunology Hematology and Rheumatology Unit, Necker University Hospital, Assistance Publique-Hôpitaux de Paris (AP-HP), Paris, France; ^2^ French National Reference Center for Primary Immune Deficiencies (CEREDIH), Necker University Hospital, Assistance Publique-Hôpitaux de Paris (AP-HP), Paris, France; ^3^ Pediatric Oncology and Hematology Unit, Children Hospital, Vandoeuvre-les-Nancy, France; ^4^ Department of Pediatric Hematology-Oncology, CHRU Lille, Lille, France; ^5^ Pediatric Immuno-Hemato-Oncology Unit, Angers University Hospital, Angers, France; ^6^ Clinical Development and Medical Affairs Unit, Scientific, Medical and Regulatory Affairs Department, Laboratoire Français du Fractionnement et des Biotechnologies (LFB), Les Ulis, France; ^7^ Pharmacovigilance Unit, Scientific, Medical and Regulatory Affairs Department, Laboratoire Français du Fractionnement et des Biotechnologies (LFB), Les Ulis, France; ^8^ Department of Pediatric Hematology and Oncology, Rouen University Hospital, Rouen, France; ^9^ Pediatric Oncology Hematology Unit, University Hospital, Bordeaux, France; ^10^ Department of Pediatric Oncology, University Hospital of Saint Etienne, North Hospital, Saint Etienne, France

**Keywords:** immunoglobulin, primary immunodeficiency, Clairyg®, post-approval safety study, real-world experience, pediatrics, immune thrombocytopenia

## Abstract

**Introduction:**

This study presents the results of a real-life, multicenter, prospective, post-approval safety evaluation of Clairyg® 50 mg/mL, a 5% intravenous immunoglobulin (IVIg) liquid, in 59 children (aged < 12 years) with primary immunodeficiency diseases (PID) (*n* = 32) or immune thrombocytopenia (ITP) (*n* = 27) in France.

**Methods:**

The primary objective of the study was to assess the safety and tolerability of Clairyg®, recording all serious and non-serious adverse events (AEs), whether related (rAEs) or not related to the product. Secondary objectives aimed at evaluating the administration of Clairyg® under routine conditions and the available efficacy data to better document the benefit/risk ratio in this pediatric population. An exploratory objective was added to evaluate the potential factors associated with the occurrence of rAEs. Patients received Clairyg® according to the approved dosage under normal conditions of prescriptions over a median follow-up period of 11.8 months.

**Results:**

A total of 549 infusions (PID: *n* = 464 and ITP: *n* = 85), were administered, of which 58.8% were preceded by premedication. The most frequent rAEs were headache, vomiting, and pyrexia in both indications. Most of them were considered non-serious and mild or moderate in intensity. A severe single rAE was observed (aseptic meningitis) in a 4-year-old girl presenting with chronic ITP. The exploratory multivariate analysis of potential co-factors showed that the occurrence of rAEs is significantly linked to high IVIg doses and possibly to female gender. The annualized rate of serious bacterial infections was 0.11 for patients with PID. For patients with ITP, 74.1% experienced at least one bleeding episode during the follow-up, mostly a cutaneous one, and none had gastrointestinal, genitourinary, or central nervous system bleeding.

**Conclusion:**

Clairyg® was well tolerated and allowed for control of serious bacterial infection in PID and serious bleeding in ITP, which are the main complications in these respective pediatric disorders. No new safety signal was detected in children less than 12 years-old in real-life conditions of use.

## 1. Introduction

Intravenous immunoglobulin (IVIg) from healthy donors is a widely used therapy for both patients with autoimmune diseases (as immunomodulatory therapy) and immunocompromised patients (as replacement therapy) (1, 2). IVIg is generally considered to have an acceptable safety profile, with most adverse reactions being mild and reversible (3). The rate of systemic adverse events in subcutaneous immunoglobulin (SCIg) recipients is described as lower than among IVIg treated patients. Nevertheless, the choice of treatment option for young children is usually based on both the physician's experience and family preference, as this can improve compliance and quality of life (1). However, despite children being considered to have a lower susceptibility to adverse events (4), there has been little research investigating IVIg safety in pediatric patients, particularly in children under 12 years of age.

Clairyg® 50 mg/mL, referred in the text as Clairyg® (LFB Biomédicaments), is a highly purified human IgG preparation that is a liquid, ready-to-use form. It was approved in France in 2009, with the commitment by the applicant to conduct a post-authorization safety study (PASS) in the pediatric population. At the time of approval, the relatively limited experience with Clairyg® in children justified extending the observation in current medical practice. The study was limited to children under 12 years of age, as it was estimated and validated by the Paediatric Committee of the European Medicines Agency that adolescents aged 12–18 years can be treated similarly to adults. This hypothesis was later confirmed by a post-pivotal long-term safety and efficacy study that reported safety data in six primary immunodeficient adolescents aged 12–18 years, as well as by the initial post-registration exposure data.

This national, prospective, observational study was conducted in children treated for either one of the two main approved indications: PID (where IVIg is used as long-term replacement therapy) and ITP (where higher IVIg dosages are used more temporarily and specifically for their immunomodulatory effects).

The primary objective of the study was to assess the safety profile of Clairyg® when administered to children under 12 years of age. Secondary objectives aimed to collect information on the general use of Clairyg® in these children, as well as any available information on its clinical efficacy, to further document the benefit/risk ratio. An exploratory analysis was also conducted to evaluate potential factors associated with the occurrence of related adverse events.

## 2. Materials and methods

### 2.1. Study design

This study was a non-interventional, prospective, multicenter, open-label post-authorization safety study (PASS) conducted in seven French centers from October 2015 to November 2017. The recruitment and follow-up periods each lasted 12 months. Patient inclusion in the study was independent from the decision to prescribe or use the product. A sponsor-independent Scientific Committee, comprising two expert pediatricians and a statistician, provided medical, scientific, and methodological guidance by reviewing the protocol and each case report form, and evaluating safety and efficacy data.

### 2.2. Study drug

Clairyg® is a saccharose- and maltose-free liquid IVIg with a 5% concentration, possessing a high biological safety profile. The product is manufactured using a purification process that includes ethanolic and caprylic precipitation steps, followed by anion-exchange and affinity chromatography steps. This results in a final product that preserves all IgG functionalities and contains low levels of IgA, IgM, as well as anti-A and anti-B hemagglutinins (5–7).

In this study, the investigators prescribed Clairyg® according to the Summary of Product Characteristics (SmPC) and their own clinical experience. For each administration, the study team collected information on the dose, duration, and infusion rate, as well as any pre-medication received.

### 2.3. Patients

The inclusion criteria for this study required patients under 12 years of age to receive treatment with Clairyg® for either a PID or ITP, regardless of the stage of their disease. Patients or their legal representatives were required to read, understand, and sign a written informed consent/assent form. As all patients were minors, at least one parent or legal representative signed the consent form. Additionally, whenever deemed appropriate by the investigator, oral information was provided to the child, who also could sign an assent form with parental authorization. No exclusion criteria were established.

### 2.4. Data collection

Upon inclusion, the study team collected data on patients' demographic characteristics, including age, gender, indication for treatment, as well as their medical history. Prospective follow-up data was then collected for 12 months in a centralized database using an electronic case report form.

### 2.5. Safety

Adverse events (AEs) and abnormal laboratory parameters were systematically collected and monitored throughout the study. The nature of AEs and serious adverse events (SAEs) were analyzed using the Medical Dictionary for Regulatory Activities (MedDRA®) System Organ Class (SOC) and Preferred Term (PT) descriptors (8). Each AE was described and analyzed according to its nature, severity, seriousness, frequency, and outcome. The investigators assessed the relationship of all AEs with Clairyg® according to the Good Pharmacovigilance Practices (9).

### 2.6. Efficacy

As part of the secondary objectives, all infections (whether severe or not, bacterial or not) occurring during the study were collected in children with PID. Additionally, data on preventive or curative antibiotics received (including type, duration, route of administration, and frequency), as well as trough plasma IgG levels before IVIg infusions (when available), were recorded. For children with ITP, all bleeding episodes occurring during the study were collected, along with data on platelet count evolution after IVIg infusions (when available). The investigators were also asked to record any cause of study discontinuation or possible lack of efficacy observed during the follow-up.

### 2.7. Statistical methods

Quantitative variables were described using means, standard deviations, and/or medians with minimum & maximum values, while qualitative variables were described using frequencies. Cohorts were considered both separately and pooled. An exploratory analysis was carried out to analyze the relationship between the occurrence of related adverse events and factors such as age, sex, diagnosis, and dose. Mixed-effects logistic regression was used to consider intra-patient correlation. Modelling started with a full multivariable model and, after a backward selection method, the model with the smallest Akaike Information Criterion was retained. Odds ratios (OR) and their associated 95% confidence intervals and *p*-values are presented.

### 2.8. Ethics

The study protocol received a positive opinion from the Expert Committee of the French Ministry of Research, which assessed the ethical and methodological aspects of the study in November 2014. The study was authorized by the French national data protection commission in July 2015, ensuring the protection of personal data in accordance with national regulations.

## 3. Results

### 3.1. Patient's demographics and disease characteristics

A total of 59 children under the age of 12 were included in the study, consisting of 32 patients with primary immunodeficiencies (16 predominantly humoral and 16 combined PID such as Wiskott-Aldrich syndrome, activated PI3K-delta syndrome, ataxia telangiectasia, CTLA-4 or PMG3 deficiency) (Supplementary Table S1) and 27 patients with ITP (17 acute and 10 non-acute including 2 persistent and 8 chronic ITP).

Of the participants, 64.4% were male, and the age range was 0.3–11.9 years, with 76.3% of participants under the age of 8 years. The median age at diagnosis was 2 years [0–10 years], and the median disease duration before inclusion was 2 years [0–11 years]. The majority of participants (71.2%) were already receiving treatment with intravenous or subcutaneous Ig (41 patients), corticosteroids (8 patients with ITP), and/or other therapies (6 patients with ITP), such as anti-CD20 monoclonal antibody, anti-D immunoglobulin, immunosuppressive agents, or thrombopoietin receptor agonists. The remaining 28.8% were treatment-naïve, with only one patient with PID not receiving any prior substitution treatment and 16 patients with ITP not receiving any immunomodulatory treatments (Table 1).

**Table 1:** Patient and disease characteristics at baseline.

		PID, *N* = 32	ITP, *N* = 27	Global, *N* = 59
Age, years	Mean (SD)	6.4 (3.3)	4.4 (3.2)	5.4 (3.4)
	Median, [range]	5.7 [0.7–11.9]	3.3 [0.3–11.7]	4.8 [0.3–11.9]
Age by category, *n* (%)	<4 years	6 (18.8)	16 (59.3)	22 (37.3)
	[4;8[	16 (50.0)	7 (25)	23 (39.0)
	[8;12[	10 (31.3)	4 (14.8)	14 (23.7)
Gender, *n* (%)	Female	9 (28.1)	12 (44.4)	21 (35.6)
	Male	23 (71.9)	15 (55.6)	38 (64.4)
BMI, kg/m^2^	Mean (SD)	16.2 (2.4)—*n* = 31	16.1 (1.5)—*n* = 23	16.2 (2.0)—*n* = 54
Age at diagnosis	Median [range] years	2.0 [0.0–10.0]	2.0 [0.0–8.0]	2.0 [0.0–10.0]
Disease duration	Median [range] years	3.0 [0.0–11.0]	0.0 [0.0–7.0]	2.0 [0.0–11.0]
Prior therapy *n* (%)	None	1 (3.1)	16 (59.3)	17 (28.8)
	Prior therapy: IVIg SCIg Corticosteroids Others^a^	31 (96.9)	11 (40.7)	42 (71.2)
		30 (96.8)	10 (90.9)	40 (67.8)
		1 (3.2)	0	1 (1.7)
		0	8 (72.7)	8 (13.6)
		0	6 (54.5)	6 (10.2)
	Splenectomy	–	1 (3.7)	1 (1.7)
Biological characteristics at baseline		Trough plasma IgG levels median: (g/L) 9.7 [2.6–13.5]	Platelet counts: *n* (%) <10 × 10^9^/L: 20 (74.1) 10–150 × 10^9^/L: 7 (25.9)	* *

BMI, body Mass index; IgG, immunoglobulin G; ITP, immune thrombocytopenia; IVIg, intravenous immunoglobulin; PID, primary immunodeficiency; SD, standard deviation; SCIg, subcutaneous immunoglobulin.

^a^ anti-CD20 monoclonal antibody, anti-D immunoglobulin, immunosuppressive agents, thrombopoietin receptor agonist.

Of the 32 patients with PID, almost 60% had complications secondary to PID, half of which were respiratory (Supplementary Table S2). Among the 27 patients with ITP, all but one presented with cutaneous (96.3%) and mucosal (73.1%) bleeds. A single patient with non-acute ITP had no bleeding at inclusion but had a platelet count of 22 × 10^9^/L.

Of the 59 participants, 37 (62.7%) had other comorbidities, the most common being respiratory (35.1%), cutaneous (21.6%), infectious (18.9%) or congenital (16.2%) (Supplementary Table S3). The most frequently used concomitant medications were antibiotics, analgesics, glucocorticoids, and bronchodilators for children with PID, and antihistamines or analgesics for children with ITP.

### 3.2. IVIg treatment

The median study participation duration was 11.8 months [0.3–12.9 months], and the median treatment duration was 197 days [1–382 days], with a total of 549 infusions administered during the study period (464 for patients with PID and 85 for patients with ITP). The median number of infusions per patient was 6 [1–24] (17.5 [1–23] for patients with PID and 1 [1–24] for patients with ITP). The median IVIg dose administered was 0.4 g/kg [0.2–1.1] for the overall population [0.4 g/kg (0.2–0.9) for patients with PID and 1 g/kg (0.4–1.1) for patients with ITP]. Most of the dosing regimens remained unchanged during the study period. In 17 patients with PID, a posology adjustment was performed due to children's growth [22/464 (4.7%) infusions], none of which were due to an adverse event. The median time between two infusions for the 41 patients who received more than one infusion was 3.1 weeks [0.3–38.3]. Whenever available [425/549 (77.4%) infusions], the median duration of infusion was 3.3 h [0.2–24.5], with a mean infusion rate of 2.3 ± 0.6 mL/kg/h (for 434 infusions).

Premedication was used in around 60% of cases, usually with acetaminophen, antihistamines, systemic or local anesthetics, glucocorticoids, or parenteral solutions for pre-hydration.

Results of each cohort are displayed in Table 2.

**Table 2:** IVIG exposure.

		PID, *N* = 32	ITP, *N* = 27	Global, *N* = 59
Length of follow-up, months	Median [range]	12.0 [0.3–12.7]	10.3 [0.6–12.9]	11.9 [0.3–12.9]
Total number of infusions		464	85	549
Treatment duration, days	Median [range]	365.5 [1–382]	1.0 [1.0–372]	197.0 [1.0–382]
Number of infusions per patient	Median [range]	17.5 [1–23]	1 [1–24]	6 [1–24]
Time between 2 infusions per patient, weeks	*n patients*	*n = 31*	*n = 10*	*n = 41*
	Median [range]	3.1 [2.4–6.0]	6.0 [0.3–38.3]	3.1 [0.3–38.3]
Dose administered per infusion, g/kg	Median [range]	0.4 [0.2–0.9]	1.0 [0.4–1.1]	0.4 [0.2–1.1]
Duration of infusion per infusion, hours	*n infusions*	*365/464*	*60/85*	*425/549*
	Median [range]	3.3 [0.2–8.0]	8.3 [0.5–24.5]	3.3 [0.2–24.5]
per infusion: Mean infusion rate, mL/kg/h Initial flow rate, mL/kg/h *Final flow rate, mL/kg/h	*n infusions*	377 or 376*/464	*57/85*	434 or 433*/549
	Median [range]	2.5 [1.0–5.0]	1.8 [0.8–3.9]	2.4 [0.8–5.0]
	Median [range] Median [range]	1 [0.9–5.0]	1 [0.4–3.9]	1 [0.4–5.0]
		*(48.8% > 1)*	*(10.5% > 1)*	*(43.8% > 1)*
		3.9 [1.1–5]	2.5 [0.8–7.1]	3.8 [0.8–7.1]
		*(9.0% > 4)*	*(1.7% > 4)*	*(8.1% > 4)*
Premedication	*n infusions (%)*	*270 (58.2)*	*53 (62.4)*	*323 (58.8)*
	*n* patients (%)	20 (62.5)	9 (33.3)	29 (49.2)

ITP, immune thrombocytopenia; IVIg, intravenous immunoglobulin; PID, primary immunodeficiency.

### 3.3. Adverse events

Of the 59 patients, 47 experienced a total of 169 adverse events (AEs), most of which were mild (81.1%) or moderate (17.8%) in intensity. Of these AEs, 44.4% (75/169) were considered possibly related to the product and occurred in 13 boys (43 AEs) and 15 girls (32 AEs). The mean incidence rate of related AEs per infusion was 0.1 ± 0.5 (0.1 ± 0.4 in boys and 0.2 ± 0.6 in girls), with the majority (89.9%) occurring within 3 days of infusion (Table 3).

**Table 3:** Patients with related adverse events (number and most common types).

	PID, *N* = 32	ITP, *N* = 27	Global, *N* = 59
Related AE, number (%)	44 (44%)	31 (44.9%)	75 (44.4%)
Patients with at least 1 rAE	15 (46.9%)	13 (48.1%)	28 (47.5%)
Number of rAE/patient			
Mean (SD)	1.4 (2.3)	1.2 (1.5)	1.3 (2.0)
Median [range]	0 [0–8]	0 [0–5]	0 [0–8]
Number of rAE/infusion	464 infusions	85 infusions	549 infusions
Mean (SD)	0.1 (0.4)	0.4 (0.7)	0.1 (0.5)
Median [range]	0 [0–4]	0 [0–4]	0 [0–4]
Serious rAE, number	0	1	1
Patient with at least 1 serious rAE	0	1 aseptic meningitis	1
Time to onset of rAE, number (%) - The day of infusion - Within 3 days - More than 3 days after	*n = 41 rAE*	*n = 28 rAE*	*n = 69 rAE*
	27 (66)	12 (43)	39 (57)
	9 (22)	14 (50)	23 (33)
	5 (12)	2 (7)	7 (10)
Outcome of rAE, number (%)			
- Recovered without sequelae - Not recovered at the end of follow-up	43 (97.7)	30 (96.8)	73 (97.4)
	1 (2.3)	1 (3.2)	2 (2.6)
Most common systemic rAE (*n* > 5%): *n* (%)			
Headache	18 (40.9)	6 (19.4)	24 (32.0)
Vomiting	8 (18.2)	5 (16.1)	13 (17.3)
Pyrexia	2 (4.5)	7 (22.6)	9 (12.0)
Pain in extremity	0	4 (12.9)	4 (5.3)

AE, adverse event; rAE, related adverse event; ITP, immune thrombocytopenia; PID, primary immunodeficiency; SD, standard deviation.

The most common related AEs were headache (32%), vomiting (17.3%), and fever (12%) (Table 3 and Supplementary Table S4). Only one related serious adverse event (rSAE), aseptic meningitis, was observed in a 4-year-old girl with chronic ITP. This SAE was partially documented, and treatment was temporarily interrupted, followed by a reduced infusion rate when reintroduced. The aseptic meningitis did not recur, but two related AEs (vomiting and headache) occurred during the third and final infusion administered during the study.

Laboratory monitoring revealed five clinically significant abnormalities, associated with three non-serious related AEs in two patients with ITP: two transient increases in blood creatinine and one decrease in hemoglobin (from 11.1 to 9.5 g/dL), reported as hemolytic anemia (but not properly documented) without clinical signs of hemolysis and possibly due to a concomitant EBV infection as the underlying cause. This was identified retrospectively by the investigator during chart review.

No thrombotic events were observed, and only one local reaction occurred in one patient with PID after an extravasation at the infusion site, requiring replacement of the infusion set. Overall, only two related AEs (2.6%) were not resolved at the end of follow-up (one peripheral edema, which later resolved, and one extremity pain).

### 3.4. Association between the occurrence of related-adverse events and other co-factors

The results of the exploratory analysis showed that high IVIg doses (OR: 10.3; 95% CI: 3.1–34.4 for IVIg ≥ 0. 8 g/kg; *p*-value < 0.0007), female gender (OR: 5.8; 95% CI: 1.8–19; *p*-value < 0.0038) and combined gender and premedication effect (girls without premedication before infusion) (OR: 4.9; 95% CI: 1.3–19.2; *p*-value < 0.0052) were significantly associated with the occurrence of related adverse events.

### 3.5. Efficacy

The primary objective of the trial was not to assess the efficacy of Clairyg®. However, the efficacy data were collected to contribute to the recurrent benefit/risk assessment of the product for pharmacovigilance purposes. Results of PID and ITP cohorts are displayed in Tables 4, 5 respectively.

**Table 4:** Efficacy data in patients with PID.

Efficacy parameters (during the study)		PID, *N* = 32
Trough plasma IgG levels (g/L)	Mean (SD)	9.98 (2.63)
	Median [min; max]	9.48 [2.2; 18.1]
Number of infections per patient	Mean (SD)	5.7 (4.1)
	Median [min; max]	5.0 [0; 18]
Patients with at least 1 infection n (%)	All type/all serious^a^	30 (93.8)/6 (18.8)
	Bacterial/serious bacterial	16 (50)/3 (9.4)
Number of episodes, *n* (%)	All type/ all serious^a^	171/8
	Bacterial/serious bacterial	53 (31)/3 (37.5)
Annual rate of infection (rate/patient/year)	All type/all serious^a^	6.20/0.29
	Bacterial/serious bacterial	1.92/0.11
Outcome of infection at the end of follow-up, *n* (%)	Resolved without sequelae	161 (94.2)
	Recovering/not recovered	1 (0.6)/8 (4.7)
	Fatal	1 (0.6)
Number of patients using antibiotics, *n* (%)	Curative antibiotics	22 (68.8)
	Prophylactic antibiotics	27 (84.4)
Lack of efficacy in the opinion of investigators, *n* (%)		0 (0)

IgG, immunoglobulin G; PID, primary immunodeficiency; SD, standard deviation.

^a^ Seriousness criteria (at least one present): results in death, life-threatening, hospitalization or prolongation of hospitalization, persistent or significant disability/incapacity, important medical event (9).

**Table 5:** Efficacy data in patients with ITP.

Efficacy parameters (during the study)		ITP, *N* = 27
Platelet count per patient (10^9^/L)	Mean (SD)	133.2 (101.0)
	Median [min; max]	108.4 [4; 359]
Patients with at least 1 episode of bleeding, *n* (%)	All type	20 (74.1)
	Cutaneous	20 (74.1)
	Mucosal	14 (51.9)
	Hemarthrosis	1 (3.7)
	Gastro-intestinal	0
	Central nervous system	0
Change in the ITP status at the end of follow-up, *n* (%)	Acute to remission	11 (40.7)
	Acute to persistent	4 (14.8)
	Acute/persistent to chronic	3 (11.1)
	No change (chronic)	8 (29.6)
	Lost to follow-up (Acute)	1 (3.7)
Number of patients using other ITP therapies, *n* (%)		11 (40)
Lack of efficacy in the opinion of investigators, *n* (%)		1 (3.7)

ITP, immune thrombocytopenia; SD, standard deviation.

#### 3.5.1. Patients with PID

The annualized rates of infections and serious bacterial infections were 6.2 and 0.11 per patient, respectively. Almost 85% of the patients received prophylactic antibiotic treatment. Only 6 out of 32 patients (less than 20%) had severe infections, and most of the infections resolved without sequelae. One patient had a fatal outcome due to pulmonary and meningeal sepsis, unrelated to Clairyg®. This patient had a severe combined PID with a PGM 3 deficiency, had undergone an allogeneic hematopoietic bone marrow stem cell transplant 8 months prior to enrolment and received a single Clairyg® infusion during the study.

The median trough plasma IgG level was 9.48 g/L [2.2–18.1], with 80% between 7 and 13.5 g/L. None of the investigators reported a lack of efficacy in the 32 patients treated, including the 7 patients who were in the study for less than 12 months (6 patients had a change in replacement therapy, and one patient died).

#### 3.5.2. Patients with ITP

No gastrointestinal, central nervous system, or gynecological-urinary system bleedings was observed in the overall population. However, in the case of a 4-year-old girl with non-acute ITP, who received her first infusion at approximately 1 g/kg, a platelet increase from 3 to 13 × 10^9^/L was observed. Due to the occurrence of aseptic meningitis, the next two consecutive infusions were given at a lower dose (0.4 g/kg). However, platelets count did not increase, and the investigator reported a lack of efficacy.

In the subgroup of patients with acute ITP, 11 out of 17 patients (64.7%) were in remission at the end of follow-up, 4 (23.5%) progressed to persistent ITP, 1 progressed to chronic ITP, and 1 was lost to follow-up. In 5 of these, corticosteroids were associated with Clairyg®. Ten (58.8%) patients had at least one cutaneous bleed, 6 (35.3%) had at least one mucosal bleed, and a single patient (3.7%) hada joint bleed (elbow hemarthrosis). The median platelet count after treatment with Clairyg® in these 17 patients with ITP was 167.4 × 10^9^/L [23–359 × 10^9^/L].

All 10 patients with non-acute ITP had at least one cutaneous bleed, and 80% had at least one mucosal bleed. The median platelet count on treatment with Clairyg® in this subgroup was 47.8 × 10^9^/L [4–154 × 10^9^/L]. Six of these 10 patients were receiving concomitant treatment with corticosteroids (4 patients), immunosuppressive agent (1 patient), vinca alkaloid (2 patients), purine analogue (1 patient), and thrombopoietin receptor agonist (1 patient).

## 4. Discussion

The main objective of this observational study was to describe the adverse events that occurred in children under 12 years of age who were treated with Clairyg® for PID or ITP over a 12-month period in real-life conditions. Secondary objectives included specifying the prescription procedures and conditions of use and describing the characteristics of the patients receiving this product, as well as their clinical and biological monitoring.

### 4.1. Patient population

Seven university hospitals agreed to participate in the study, with two centers participating in the recruitment of PID patients only. The sites were distributed throughout France equally covering regions with either high or low prevalence for both PID and ITP indications, according to published data (10, 11).

The study successfully achieved its objective of recruiting at least 25 PID patients under 12 years of age, with a total of 32 patients enrolled, of whom 25 will be followed up for more than 12 months. The 32 patients had a mix of predominantly antibody or combined immunodeficiencies, illustrating the spectrum of the disease. In contrast to what is commonly described in publications on PID (10, 12), our cohort included a high proportion of patients with combined deficiencies, such as Wiskott-Aldrich syndrome, activated PI3K-delta syndrome (APDS), ataxia telangiectasia, CTLA-4, or PMG3 deficiency. In all cases, the indication for IVIG treatment was justified, as all patients had a clinically and biologically eligible humoral deficiency for IVIg prescription, as recommended by current guidelines (13).

To our knowledge, only four exclusively pediatric PID studies have been published in the last 10 years, all reporting on children and adolescents (<17 years) with common variable immunodeficiency or X-linked agammaglobulinemia (14–17). Of these, three studies used IVIg 10% and only one used IVIg 5% use. Our study, therefore, provides informative data on the use of IVIg 5% in children with combined immunodeficiencies.

Recruitment of children with ITP proved to be more challenging than expected, with only 27 patients of the planned 30 patients enrolled. Nonetheless, the 27 patients enrolled had a wide range of clinical variability, with 17 patients having acute ITP and 10 patients having non-acute ITP. This illustrates the diverse phenotypic spectrum of patients with ITP treated in general pediatric units. Once again, the literature on ITP in children under the age of 12 years is limited, with only three prospective pediatric studies published on this topic in the last 10 years. However, all three studies described children and adolescents (<20 years) with acute or chronic ITP (18–20).

### 4.2. Clairyg® dosing regimen in real-life

The dosing regimen for Clairyg® in real-life conditions was consistent with the product's SmPC in terms of dose and frequency of administration. However, compliance with the recommended infusion rates and levels was more difficult to verify, as this was not always recorded in the patient files. In some centers, infusions were given at the same rate for all patients, mainly in those whose first IVIg infusions were well-tolerated (7/32 PID and 6/27 ITP). Although infusion rates can vary among different IVIg products (up to 8 mL/kg/h for some products), it was reassuring to observe that prescriptions for Clairyg® were adequate, with only a few infusions administered at a rate above 4 mL/kg/h (not exceeding 5 mL/kg/h for patients with PID and up to 7.1 mL/kg/h for one patient with ITP) without any specific adverse events being reported.

In addition to the dose and infusion rate, the need for special monitoring during the first infusion was appropriately followed by the prescribing centers. Due to higher doses administered, patients with ITP had a longer infusion time than patients with PID (8.3 vs. 3.3 h), with an even greater difference observed between patients with acute and chronic ITP (12.7 vs. 6.9 h respectively).

### 4.3. Real-life efficacy of Clairyg® in PID cohort

In addition to assessing real-world utilization, this study evaluated the efficacy of IVIg treatment as a secondary objective in the two cohorts. Among patients with PID, the annualized incidence rates of infections and serious bacterial infections per patient were consistent with those reported in other studies (serious bacterial infections ranging from 0 to 0.12) (14, 17). As the primary goal of IVIg administration is to prevent recurrent, severe, or unusual infections, rather than the common infections frequently observed in children without immunological deficiencies (21), the finding that 93.8% of the children experienced at least one infection (mostly viral and non-serious) during a one-year follow-up period is consistent with previously published rates in this age group (ranging from 90% to 100%) (14, 17). Furthermore, the trough plasma IgG levels (median 9.5 g/L) in our cohort were higher than the recommended minimum threshold between infusions (≥5–6 g/L), likely reflecting a deliberate decision by the investigators based on the clinical severity of individual patients, particularly those with combined immunodeficiencies (ID). Trough plasma IgG levels remained stable throughout the study, with only three patients presenting with a level below this recommendation once during their follow-up, but remaining clinically stable. Finally, the high proportion of children with combined ID, who are at a higher risk of serious infections, and the recommendation by French experts to initiate prophylactic anti-infective treatment in children with PID prone to chronic infections (22), explain the high number of patients receiving prophylactic antibiotic treatment (84.4%). In this cohort, 100% of children with combined ID and almost 70% of those with predominantly antibody deficiency received anti-infective prophylaxis with a sulfonamide, macrolide, or penicillin.

### 4.4. Real-life efficacy of Clairyg® in ITP cohort

The analysis of efficacy data in patients with ITP has been challenging due to the diverse clinical scenarios treated and the presence of acute and non-acute disease stages. The decision to initiate treatment in ITP is primarily based on the platelet count level, which varies according to the type and stage of the disease. In children, acute thrombocytopenia can spontaneously resolve, but IVIg administration can rapidly increase the platelet count to a sufficient level (>20–30 × 10^9^/L) to prevent serious bleeding events (23, 24). This study demonstrated the ability of Clairyg® to increase platelet counts (median: 167.4 × 10^9^/L [range: 23–359] for acute ITP and 47.8 × 10^9^/L [range: 4–154] for non-acute ITP), which was effective in preventing significant bleeding as there were no gastrointestinal, genitourinary, or central nervous system bleedings. Additionally, the occurrence of mucosal bleeding after at least one infusion was lower in patients with acute ITP (35.3%) than non-acute ITP (80%), which was correlated clinically with the respective platelet count thresholds achieved. Early use of IVIg is believed to lower the risk of chronic ITP (23). During the 12-month follow-up period, chronic ITP was observed in only one newly diagnosed child (5.9%), and four (23.5%) had persistent ITP. These results are comparable to the previously published 18% conversion rate to chronic ITP (24), although comparisons remain difficult as the efficacy was evaluated at six months (compared to 12 months in our study), and specific criteria related to complete or partial responses were used (which were not utilized in this study).

### 4.5. Pooled PID/ITP safety data

The primary objective of this study was to describe the safety profile of our product in real-world settings. Pooled safety data from the two cohorts indicated a favorable safety profile for children with both primary immunodeficiency and immune thrombocytopenia, complementing routine pharmacovigilance data obtained from spontaneous reports by healthcare professionals since market access.

The reported rate of adverse events following IVIg infusion varies widely in the literature (from 1 to 80% of patients, depending on product use) (3), but children are generally considered to be less susceptible to AEs (4). Overall, 44.4% of children in this study experienced at least one related AE during follow-up, which is comparable to the rate of 44.8% observed in a prospective pediatric study focusing on immediate and delayed AE following IVIg (4). The majority of the reported AEs were mild to moderate in intensity (98.9%) and reversible (97.4%) in both immuno-replacement and immunomodulation scenarios. They consisted mainly of headaches, vomiting, and fever, which is consistent with the most commonly reported AEs associated with other products (14–20).

This study also documented the time to onset of related adverse events. Adverse reactions can be immediate (60%) or delayed (25), as confirmed in this cohort, with most symptoms occurring on the day of infusion (57%). This delay is consistent with the symptoms observed, which for immediate related AEs are mainly due to complement activation (by aggregates, kallikreins, or stabilizing agents present in IVIg preparations), such as flu-like syndromes (including headache, nausea, fever, asthenia, cough, etc.) or dermatological symptoms (eczema) (3). No events related to anaphylactic reactions or thrombosis were observed, indicating a well-controlled fractionation process of the product. With regard to delayed reactions, such as migraine (2.7%) or aseptic meningitis (1.3%), the later onset may be explained by the need to cross the blood-brain barrier (26). The rate of such reactions in our study was low, and again in line with previously reported rates (approximately 1% for the aseptic meningitis according to retrospective studies) (3, 27). Finally, no new safety signals were identified for children, suggesting that the product has a consistent and favorable safety profile.

Immediate reactions to IVIg infusions are known to be associated with rapid infusion rates (3), and it is recommended to start the infusion slowly and increase the rate gradually. This association was observed in our study, as two children with PID who received infusions at a single rate above 4 mL/kg/h experienced episodes of headache, vomiting, or pollakiuria. In addition, one patient with ITP who received an infusion at an initial rate greater than 1 mL/kg/h (at 1.9 mL/kg/h) had to stop temporarily the infusion due to nausea, fever, and cough. Resuming the infusion at a lower rate (0.9 mL/kg/h) was well tolerated with no recurrence of symptoms. These findings highlight the importance of following the low-rate recommendations as displayed in the SmPC.

Premedication is often used to alleviate these related AEs, and while it has been shown to significantly reduce their incidence, it does not eliminate them entirely (4). In routine practice, acetaminophen, antihistamines, or steroids are commonly used, as reported by 45% of patients in a survey of 1,500 patients treated with IVIg (3). Pre-hydration and good hydration during the IVIg infusion are also on the list of recommendations to prevent delayed or late events especially aseptic meningitis, renal impairment or thrombotic events in high-risk patients (3). Consistent with this, approximately 50% of children in our study received premedication (including parenteral hydration), especially those receiving chronic IVIg treatment (59% of PID infusions and 77% of non-acute ITP infusions).

### 4.6. Factors of interest associated to the occurrence of related-adverse events

Limited clinical data is available on predictive risk factors for related AEs in children. Previous studies have suggested that IVIg preparations, dosage, infusion rates, primary infusion, pre- or post-infusion hydration, underlying disease, and age may all contribute to the occurrence of AEs (3, 28). Our study confirmed that dose is a significant factor, as higher doses (≥0.8 g/kg) were associated with a higher likelihood of rAE occurrence. Unfortunately, we were unable to examine the relationship with infusion rate due to missing data for some patients.

This observational study has limitations and remains descriptive. Some studies have suggested that female gender could be a risk factor for hemolysis or aseptic meningitis (29, 30). The girls in this trial who did not receive premedication before the immunoglobulin infusion had a higher risk of adverse reactions. The interpretation of this association, if it exists, is unclear.

However, the study accurately reflects current medical practice, and the inclusion of patients with different types of PID or ITP, regardless of time since diagnosis, ensures a representative sample of the actual clinical situations encountered in these indications.

### 4.7. Conclusion

In summary, this real-life study confirmed that Clairyg® is a safe treatment option for children under 12 years of age with either PID or ITP. The study showed that the infusions of Clairyg® helped prevent serious infections in patients with PID and bleeding in patients with ITP, which are significant concerns in this age group. The results highlight the importance of complying with the product's SmPC recommendations for the dose and infusion rate and identifying potential risk factors prior to treatment to assess the need for premedication. To further improve the quality of life of these pediatric patients with rare diseases, the tailored IVIg treatment in routine practice, with more individualized safety monitoring, may be the next crucial step.

## Data Availability

The raw data supporting the conclusions of this article will be made available by the authors, without undue reservation.
