# Supplementary material for: Real-world experience with CLAIRYG® 50 mg/mL (intravenous immunoglobulin) in children under 12 years with primary immunodeficiency or immmune thrombocytopenia: a post-approval safety study
Source: Front Pediatr. 2023 Oct 2;11:1260296. doi: 10.3389/fped.2023.1260296 (PMC10577179; doi:10.3389/fped.2023.1260296)
Supplement: Supplementary file 1 [file Table1.pdf]

List of Supplementary data Tables

Supplementary data Table 1: Types of PID

Supplementary data Table 2: Types of organ failure or complication related to PID at baseline

Supplementary data Table 3: Ongoing associated or intercurrent illnesses at baseline

Supplementary data Table 4: All types of related Adverse Events

Supplementary data Table 1: Types of PID

**Classification**	**Types of PID**	**Patients,** n (%)
**Predominantly antibody deficiencies** (n=16 ; 50%)	X-linked agammaglobulinemia (Bruton disease)	5 (15.6)
	CVID	4 (12.5)
	Hypogammaglobulinemia	4 (12.5)
	Class-switch ID	1 (3.1)
	Others	2 (6.25)
**Combined immunodeficiencies** (n=16 ; 50%)	Wiskott Aldrich syndrome	4 (12.5)
	APDS	2 (6.25)
	CTLA-4 deficiency	1 (3.1)
	STAT3 loss of function*	1 (3.1)
	SMARCD2 deficiency	1 (3.1)
	SCIDX-1	1 (3.1)
	PGM 3 deficiency	1 (3.1)
	Ataxia Telangiectasia	1 (3.1)
	Others	4 (12.5)

*APDS: Activated PI3K-delta syndrome, CIVD: Common Variable Immune deficiency, ID: Immunodeficiency, PID: Primary Immunodeficiency, SCIDX: X-linked severe combined immune deficiency, *AD STAT3 Dominant negative deficiency (AKA Job's syndrome)*

Supplementary data Table 2: Types of organ failure or complication related to PID at baseline

	**PID, N=32**
**Number of patients,** n (%)	19 (59.4)
**Respiratory types,** n (%)	**9 (47.4)**
Bronchiectasis	4 (21.1)
Chronic bronchorrhea	3 (15.8)
Other respiratory disorders	5 (26.3)
**Digestive type,** n (%)	**2 (10.5)**
Chronic diarrhea	2 (10.5)
**Other types**	**10 (52.6)**
Epistaxis	3 (15.8
Bruising of the lower limbs	3 (15.8
Eczema	2 (10.5)
Chronic conjunctivitis and oral candidiasis	2 (10.5)
Pericarditis	1 (5.3)
Multiple lymphadenopathy	1 (5.3)
Exocrine pancreatic insufficiency	1 (5.3)
Lymphoedema	1 (5.3)
Failure to thrive	1 (5.3)

PID: Primary Immunodeficiency

Supplementary data Table 3: Ongoing associated or intercurrent illnesses at baseline

MedDRA System Organ Class / Preferred Term [8]	Number of patients with PID or ITP and with at least one relevant medical or surgical history ongoing (N=37)
**Respiratory, thoracic and mediastinal disorders,** n (%)	**13 (35.1)**
Asthma	6 (16.2)
Epistaxis	2 (5.4)
Tonsillar hypertrophy	2 (5.4)
Lung disorder	1 (2.7)
Obstructive airway disorder	1 (2.7)
Productive cough	1 (2.7)
**Skin and subcutaneous tissue disorders,** n (%)	**8 (21.6)**
Eczema	2 (5.4)
Urticaria	2 (5.4)
Dermatitis diaper	1 (2.7)
Dry skin	1 (2.7)
Eczema infantile	1 (2.7)
Skin lesion	1 (2.7)
**Infections and infestations,** n (%)	**7 (18.9)**
Bronchiolitis	1 (2.7)
Ear infection	1 (2.7)
Epstein-Barr virus infection	1 (2.7)
Nasopharyngitis	1 (2.7)
Rhinitis	1 (2.7)
Tooth abcess	1 (2.7)
Tracheitis	1 (2.7)
Varicella	1 (2.7)
**Congenital, familial and genetic disorders**, n (%)	6 (16.2)
Phimosis	2 (5.4)
Atrial septal defect	1 (2.7)
Glucose-6-Phosphate Dehydrogenase deficiency	1 (2.7)
Hemoglobin disease	1 (2.7)
Ichthyosis	1 (2.7)
Renal dysplasia	1 (2.7)
**Blood and lymphatic system disorders**, n (%)	5 (13.5)
Anemia	2 (5.4)
Eosinophilia	1 (2.7)

MedDRA System Organ Class / Preferred Term [8]	Number of patients with PID or ITP and with at least one relevant medical or surgical history ongoing (N=37)
Iron deficiency anemia	1 (2.7)
Lymphadenopathy	1 (2.7)
**Gastrointestinal disorders**, n (%)	5 (13.5)
Constipation	2 (5.4)
Abdominal pain	1 (2.7)
Diarrhea	1 (2.7)
Gastro-esophageal reflux disease	1 (2.7)
**Immune system disorders**, n (%)	4 (10.8)
Food allergy	2 (5.4)
Allergy to animal	1 (2.7)
Atopy	1 (2.7)
Milk allergy	1 (2.7)
**Musculoskeletal and connective tissue disorders**, n (%)	3 (8.1)
Arthralgia	1 (2.7)
Knee deformity	1 (2.7)
Synovial cyst	1 (2.7)
**Nervous system disorders**, n (%)	3 (8.1)
Headache	2 (5.4)
Paresthesia	1 (2.7)
**Metabolism and nutrition disorders,** n (%)	2 (5.4)
Lactose intolerance	1 (2.7)
Vitamin D deficiency	1 (2.7)
**Eye disorders,** n (%)	1 (2.7)
Myopia	1 (2.7)
**General disorders and administration site conditions**, n (%)	1 (2.7)
Pyrexia	1 (2.7)
**Renal and urinary disorders**, n (%)	1 (2.7)
Hypertonic bladder	1 (2.7)

ITP: Immune thrombocytopenia, PID: Primary immunodeficiency

Supplementary data Table 4: All types of related Adverse Events

**Type of rAEs**	**PID** *(15 patients)* **n=44 rAE**	**ITP** *(13 patients)* **n=31 rAE**	**Global** *(28 patients)* **n=75 rAE**
**Local**: Extravasation at the injection site, n (%)	1 (2.3)	0	1 (1.3)
**Systemic**: n (%)			
Headache	18 (40.9)	6 (19.4)	24 (32.0)
Vomiting	8 (18.2)	5 (16.1)	13 (17.3)
Pyrexia	2 (4.5)	7 (22.6)	9 (12.0)
Pain in extremity	0	4 (12.9)	4 (5.3)
Asthenia	1 (2.3)	2 (6.5)	3 (4.0)
Migraine	2 (4.5)	0	2 (2.7)
Nausea	1 (2.3)	1 (3.2)	2 (2.7)
Blood creatinine increased	0	2 (6.5)	2 (2.7)
Pollakiuria	2 (4.5)	0	2 (2.7)
Cough	1 (2.3)	1 (3.2)	2 (2.7)
Dizziness	1 (2.3)	0	1 (1.3)
Presyncope	1 (2.3)	0	1 (1.3)
Abdominal pain	1 (2.3)	0	1 (1.3)
Hyperthermia	0	1 (3.2)	1 (1.3)
Peripheral oedema	1 (2.3)	0	1 (1.3)
Eczema	1 (2.3)	0	1 (1.3)
Hyperhidrosis	1 (2.3)	0	1 (1.3)
Hemolytic anemia	0	1 (3.2)	1 (1.3)
Aseptic meningitis (*serious)*	0	1 (3.2)	1 (1.3)
Device occlusion	1 (2.3)	0	1 (1.3)
Hot flush	1 (2.3)	0	1 (1.3)

ITP: Immune thrombocytopenia, PID: Primary immunodeficiency, rAE: related adverse event
